# Supplementary material for: Identification of photoperiod-induced specific miRNAs in the adrenal glands of Sunite sheep (Ovis aries)
Source: Front Vet Sci. 2022 Jul 22;9:888207. doi: 10.3389/fvets.2022.888207 (PMC9354845; doi:10.3389/fvets.2022.888207)
Supplement: Supplementary file 1 [file Data_Sheet_1.ZIP › Supplementary file/miRDeep2 file.zip!/miRDeep2 file/Know_Structure/LP42A2/oar-mir-382.pdf]

miRBase precursor : oar-mir-382
Total read count : 3235
oar-miR-382-5p read count : 1217
oar-miR-382-3p read count : 1996
remaining reads : 22

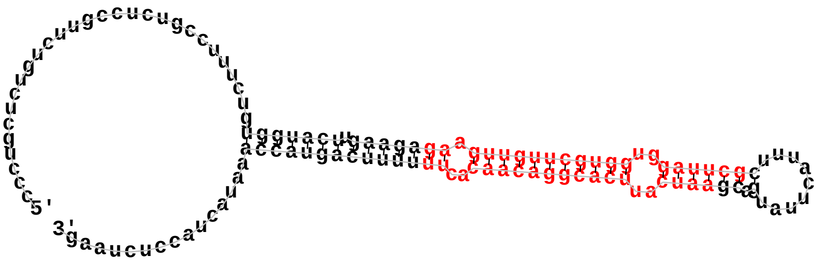

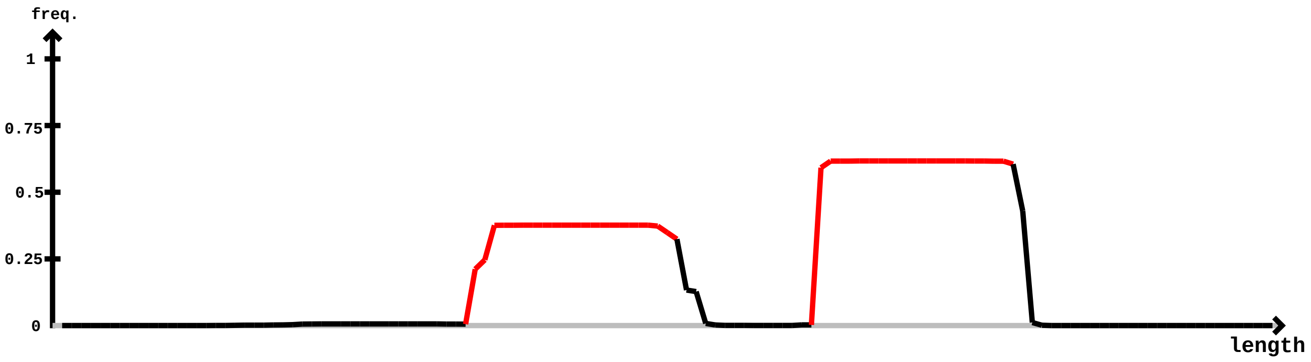


```
                                                                                    oar-miR-382-3p
                                         oar-miR-382-5p
5'- cccugcucugucuugccucugccuuucugugguacuugaagagaaguuguucgugguggauucgcuuuacuuaugacgaaucauucacggacaacacuuuuuucaguaccaaauacuaccucuaag  -3'   exp
    ....((........)).............(((((((.(((((((.(((((((((((..(((((((.........).))))))..)))))))))))..))))))))))))))...............   reads  mm
    ...............ccucugccuuucugugguacuuga.......................................................................................   1      0
    .................ucugccuuucugugguacuuga.......................................................................................   1      0
    .................ucugccuuucugugguacuugaaga....................................................................................   1      0
    ..................cugccuuucugugguacuugaaga....................................................................................   2      0
    .....................ccuuucugugguacuugaaga....................................................................................   2      0
    ......................cuuucugugguacuugaaga....................................................................................   1      0
    .......................uuucugugguacuugaag.....................................................................................   1      0
    .......................uuucugugguacuugaaga....................................................................................   2      0
    ........................uucugugguacuugaagG....................................................................................   1      1
    ........................uucugugguacuugaaga....................................................................................   6      0
    .........................ucugugguacuugaaga....................................................................................   1      0
    ..........................cugugguacuugaagaga..................................................................................   1      0
    ..........................................gaaguuguucgugguggau.................................................................   11     0
    ..........................................gaaguuguucgugguggauu................................................................   79     0
    ..........................................gaaguuguucgugguggauuc...............................................................   77     0
    ..........................................gaaguuguucgugguggauucg..............................................................   508    0
    ..........................................gaaguuguucgugguggauucgc.............................................................   8      0
    ...........................................aaguuguucgugguggauuc...............................................................   3      0
    ...........................................aaguuguucgugguggauucg..............................................................   104    0
    ...........................................aaguuguucgugguggauucgc.............................................................   6      0
    ............................................aguuguucgugguggauucg..............................................................   5      0
    ............................................aguuguucgugguggauucgc.............................................................   5      0
    ............................................aguuguucgugguggauucgcu............................................................   386    0
    ............................................aguuguucgugguggauucgcuu...........................................................   19     0
    ............................................aguuguucgugguggauucgcuuu..........................................................   5      0
    .............................................guuguucgugguggauucgcu............................................................   1      0
    ...............................................uguucgugguggauucgcuuuacu.......................................................   1      0
    ..................................................................uuacuuaugacgaaucauucacggacaa................................   1      0
    ............................................................................cgaaucauucacggacaacacu............................   1      0
    ............................................................................cgaaucauucacggacaacacuu...........................   6      0
    ..............................................................................aaucauucacggacaaca..............................   2      0
    ..............................................................................aaucauucacggacaacacu............................   35     0
    ..............................................................................aaucauucacggacaacacuu...........................   554    0
    ..............................................................................aaucauucacggacaacacuuu..........................   1291   0
```

```
                                                                              oar-miR-382-3p
                                          oar-miR-382-5p
cccugcucugucuugccucugccuuucugugguacuugaagagaaguuguucgugguggauucgcuuuacuuaugacgaaucauucacggacaacacuuuuuucaguaccaaauacuaccucuaag

..............................................................................aaucauucacggacaacacuuuu.........................   24   0
..............................................................................aaucauucacggacaacacuuuuu........................   1    0
...............................................................................aucauucacggacaacacuu...........................   8    0
...............................................................................aucauucacggacaacacuuu..........................   60   0
...............................................................................aucauucacggacaacacuuuu.........................   9    0
...............................................................................aucauucacggacaacacuuuuu........................   3    0
..................................................................................auucacggacaacacuuu..........................   2    0
```
